# 

**Published:** 1904-09

**Authors:** 


SEPTEMBER, 1904.

Vol. XXII. No. 85.

LIBRARY SURG. GEN'L'S OFFICE OCT. 3--1904

### THE BRISTOL MEDICO-CHIRURGICAL JOURNAL

*PUBLISHED UNDER THE AUSPICES OF*

*THE BRISTOL MEDICO-CHIRURGICAL SOCIETY.*

*Editor:* R. SHINGLETON SMITH, M.D., B.Sc.

WITH WHOM ARE ASSOCIATED

J. MICHELL CLARKE, M.A., M.D.,
J. LACY FIRTH, M.S., M.D., JAMES SWAIN, M.S., M.D.
P. WATSON WILLIAMS, M.D., *Assistant Editor.*
*Editorial Secretary:* JAMES TAYLOR.

*Bath* ... ... Edward J. Cave, M.D.
*Cardiff* ... ... D. R. Paterson, M.D., C.M.
*Cheltenham* ... E. T. Wilson, M.B.
*Exeter* ... ... W. Gordon, M.A., M.D.
*Gloucester* ... Rayner W. Batten, M.D.
*Newport* ... A. Garrod Thomas, M.D., C.M.
*Plymouth* . Paul Swain, F.R.C.S.
*Swansea* ... E. Le Cronier Lancaster, B.A., M.B., B.Ch.
*Swindon* ... J. Campbell Maclean, M.B., C.M.
*Weston-s.-Mare* R. Roxburgh, M.B.

BRISTOL: J. W. ARROWSMITH.

London: J. & A. Churchill, 7 Great Marlborough Street.

*Price One Shilling and Sixpence.*

